# Esophageal squamous cell carcinoma and prognosis in Taiwan

**DOI:** 10.1002/cam4.1499

**Published:** 2018-07-25

**Authors:** Ya‐Fu Cheng, Hui‐Shan Chen, Shiao‐Chi Wu, Heng‐Chung Chen, Wei‐Heng Hung, Ching‐Hsiung Lin, Bing‐Yen Wang

**Affiliations:** ^1^ Division of Thoracic Surgery Department of Surgery Changhua Christian Hospital Changhua Taiwan; ^2^ Department of Health Care Administration Chang Jung Christian University Tainan Taiwan; ^3^ Institute of Health and Welfare Policy National Yang‐Ming University Taipei Taiwan; ^4^ Division of Chest Medicine Department of Internal Medicine Changhua Christian Hospital Changhua Taiwan; ^5^ School of Medicine Chung Shan Medical University Taichung Taiwan; ^6^ School of Medicine Kaohsiung Medical University Kaohsiung Taiwan; ^7^ Institute of Genomics and Bioinformatics National Chung Hsing University Taichung Taiwan

**Keywords:** age, Esophageal cancer, prognostic factors sex, treatment

## Abstract

The prognosis of esophageal squamous cell carcinoma is poor. In order to find out appropriate treatment for each group of patients, we aim to examine the prognostic factors influencing survival for esophageal cancer patients in Taiwan. Data were obtained from the Taiwan Society of Cancer Registry. There were 14,394 esophageal cancer patients analyzed between 2008 and 2014 in this retrospective review. The impact of the clinicopathologic factors on overall survival was assessed. The following clinic‐pathologic factors were included to analyses: age, sex, tumor location, tumor length, histologic grade, clinical T, clinical N, clinical M, clinical stage, and all therapeutic methods within 3 months after diagnosis. The 5‐year survival rate was 16.8%, with a median survival of 343 days. The distribution of patients by their clinical stage is as follows: stage 0 (*n *=* *162; 1.1%); stage I (*n *=* *964; 6.7%); stage II (*n *=* *2392; 16.6%); stage III (*n *=* *6636; 46.1%); and stage IV (*n *=* *3661; 25.4%). In the multivariate analysis, age, sex, tumor location, tumor length, clinical T, clinical N, clinical M, and treatment remained independent prognostic factors. Our data indicated that age, sex, tumor location, tumor length, clinical T, clinical N, clinical M, and treatment remained independent prognostic factors. Patients who could receive surgery had significantly better outcomes.

## Introduction

Esophageal cancer is identified as the fifth leading cause of cancer death in males worldwide. In 2012, an estimated 455,800 new esophageal cancer cases and 400,200 deaths were reported 1, 2. Northern Iran, Central Asia, and North‐Central China were the high‐risk area, and 90 percent of cases are squamous cell carcinomas (SCC) 3, 4.

In Taiwan, esophageal cancer is reported to be the fifth most common cancer among males and the ninth leading cause of cancer death in 2015 with a mortality rate of 5.1 deaths per 100,000 people. Several studies have reported that foods containing N‐nitroso compounds, hot drinks, red meat intake, alcohol, and tobacco are the main risk factors for esophageal cancer 5, 6, 7, 8, 9, 10, 11.

Most patients are diagnosed at an advanced stage. For these patients, limited therapies are available and their outcomes were poor. The 5‐year overall survival (OS) rate is less than 20%, and most patients die within 1 year of diagnosis.

In order to choose adequate treatment modalities, the identification of prognostic factors is important. Chen et al. 12 reviewed the outcomes of esophageal cancer in Taiwan during 1998 to 2007 according to the American Joint Committee on Cancer (AJCC) Cancer Staging Manual, 6th edition. The application of the 7th AJCC staging system results in a better prognostic stratification of overall survival compared to the 6th edition 13, 14.

For this study, we obtained data from the Taiwan Society of Cancer Registry (TSCR) over a 6‐year period in order to find out appropriate treatment for each group of patients, and we aimed to use multivariate analysis to examine the prognostic factors based on 7th edition staging system influencing survival for esophageal cancer patients in Taiwan.

## Patients and Methods

### Database

The population data were obtained from the TSCR. The data include Taiwan's entire population of 23 million people; many researchers have used it in published studies. The database provides registration files and original claims data for each patient. All the patients were confirmed by tissue diagnosis. The study was exempt from full review by the Internal Review Board in our hospital due to the released information being used strictly for research purposes. The IRB number of our study is 171116. This study included the following items: age, sex, tumor location, tumor length, histologic grade, clinical T, clinical N, clinical M, clinical stage, and treatment. To evaluate the clinical stage, the National Health Insurance of Taiwan covered all pretreatment staging work‐ups, including upper GI endoscopy and biopsy, chest and abdominal computed tomography (CT) with oral and IV contrast, pelvic CT with contrast, positron emission tomography/computed tomography scans, and endoscopic ultrasound. The histologic confirmation was described according to the World Health Organization classification. Every observation was staged according to the 7th edition of the TNM staging system published in 2010. A patient's initial treatment is defined as the therapy administered to the patient within 3 months of diagnosis.

### Study sample

This study searched data from the TSCR between 2008 and 2014. We identified patients who were diagnosed with esophageal cancer by the diagnostic codes (C15.0, C15.1, C15.2, C15.3, C15.4, C15.5, C15.8, and C15.9) and the morphology codes (8052, 8070, 8071, 8072, 8073, 8074, 8076, 8077, 8083, and 8084). A total of 14,394 patients with esophageal cancer were identified.

### Statistical analysis

The outcome measures for our study were 5‐year overall (OS) survival rate and median survival times. The OS was calculated as the time from tissue confirmation of malignancy to either death or December 2015. This study was censored in December 2015 when the patients were alive or had died from other causes. Survival curves were plotted by the Kaplan–Meier method, and the difference in survival was calculated by the log‐rank test. Univariate and multivariate analyses were performed with the Cox proportional hazards model using SAS software. Date and cause of death were confirmed with a Taiwan death certificate database, which updated data in December 2015.

The following clinic‐pathologic factors were included to analyses: age, sex, tumor location, tumor length, histologic grade, clinical T, clinical N, clinical M, clinical stage, and all therapeutic methods within 3 months after diagnosis. We used the SAS software (SAS System for Windows, version 9.2; SAS Institute, Cary, NC) to perform the statistical analysis for this study. Statistical analysis with a *P* value <0.05 was considered statistically significant.

## Results

**Figure 1:**
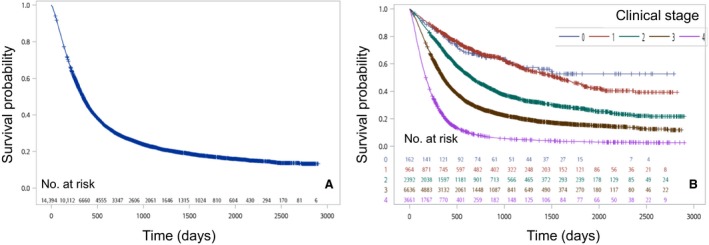
(A) Kaplan–Meier survival curves for all patients. (B) Kaplan–Meier survival curves for patients stratified by clinical stage (*P* < 0.0001).

In this study, data from 14,394 esophageal cancer patients were analyzed; 94.2% of the patients were men (*n *=* *13,558). The 5‐year OS rate was 16.8%, and the median survival time was 343 days (Fig. 1A). The clinic‐pathologic characteristics are shown in Table 1. The 5‐year OS rate was assessed and stratified according to each clinical parameter (age, sex, tumor location, tumor length, histologic grade, clinical T, clinical N, clinical M, clinical stage, and treatment method).

**Table 1:** Patient demographic data and univariate survival analysis

Variables	Numbers	5‐Year Survival (mean ±SD)	Median survival time (days, 95% C.I.)	*P* value
All	14394	16.83 ± 0.004%	343 (335 ‐350)	
Age				
<45	1547	17.09 ± 0.012%	347 (330‐374)	<0.0001
45‐64	9271	17.86 ± 0.005%	361 (352‐374)	
≧65	3576	14.09 ± 0.007%	283 (268‐298)	
Sex				
Male	13,558	16.43 ± 0.004%	341 (332‐349)	0.0003
Female	836	23.11 ± 0.017%	391 (346‐464)	
Tumor location				
L/3	3213	19.13 ± 0.008%	364 (348‐382)	<0.0001
M/3	5028	18.35 ± 0.007%	368 (354‐385)	
U/3	2897	15.04 ± 0.008%	329 (311‐347)	
Unknown	3256	13.74 ± 0.007%	295 (284‐315)	
Tumor length				
<5 cm	4360	27.03 ± 0.008%	588 (560‐624)	<0.0001
≧5 cm	5485	13.24 ± 0.006%	286 (276‐296)	
Unknown	4549			
Grade				
G1	319	21.79 ± 0.027%	439 (350‐513)	<0.0001
G2	6884	19.69 ± 0.006%	374 (360‐386)	
G3/G4	2979	13.20 ± 0.008%	332 (315‐349)	
Unknown	4212	14.45 ± 0.006%	305 (291‐317)	
Clinical T				
Tis /1	1446	40.95 ± 0.018%	1304 (1167‐1503)	<0.0001
2	1932	24.18 ± 0.012%	521 (484‐571)	
3	7035	15.19 ± 0.005%	348 (339‐355)	
4	3135	5.66 ± 0.005%	191 (182‐199)	
Unknown	846	14.77 ± 0.013%	243 (217‐272)	
Clinical N				
0	3256	30.84 ± 0.010%	684 (653‐739)	<0.0001
1	5587	14.86 ± 0.006%	339 (326‐349)	
2	2968	14.94 ± 0.009%	303 (289‐317)	
3	2025	4.38 ± 0.008%	206 (196‐217)	
Unknown	558	11.12 ± 0.014%	213 (191‐241)	
Clinical M				
0	10,753	21.32 ± 0.005%	444 (430‐459)	<0.0001
1	3417	2.99 ± 0.003%	166 (159‐173)	
Unknown	224	18.30 ± 0.026%	358 (262‐425)	
Clinical Stage				
0	162	52.70 ± 0.049%		<0.0001
1	964	44.22 ± 0.022%	1559 (1368‐1764)	
2	2392	27.48 ± 0.011%	653 (616‐692)	
3	6636	15.58 ± 0.006%	353 (343‐365)	
4	3661	3.36 ± 0.003%	171 (164‐179)	
Unknown	579	20.57 ± 0.018%	380 (325‐421)	
Treatment				
CCRT	6614	10.60 ± 0.005%	293 (286‐302)	<0.0001
CCRT + surgery	1964	29.85 ± 0.013%	707 (661‐770)	
Only surgery	1558	44.82 ± 0.016%	1446 (1272‐1649)	
Surgery + adjuvant	1071	27.55 ± 0.016%	640 (580‐695)	
Others	2010	4.63 ± 0.006%	150 (140‐160)	
Unknown	1177	5.18 ± 0.007%	105 (95‐113)	

CI, confidence interval; M,  metastasis; *N*,  node; T, tumor; SD, standard deviation; SqCC, squamous cell carcinoma.

The distribution of patients by clinical stage is as follows: stage 0 (*n *=* *162; 1.1%); stage I (*n *=* *964; 6.7%); stage II (*n *=* *2392; 16.6%); stage III (*n *=* *6636; 46.1%); and stage IV (*n *=* *3661; 25.4%). The survival curves according to clinical stage are shown in Figure 1B. The 5‐year survival rates by clinical stage were 52.7% for stage 0, 44.2% for stage I, 27.5% for stage II, 15.6% for stage III, and 3.4% for stage IV. The difference in survival was significant between neighboring stages.

**Figure 2:**
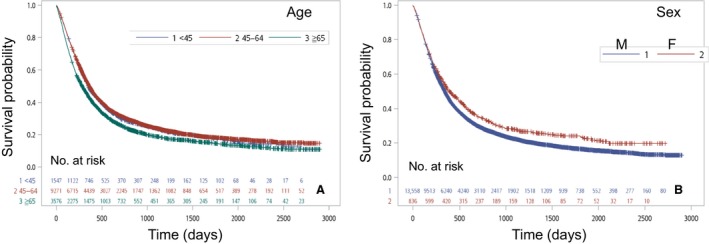
(A) Kaplan–Meier survival curves for patients according to age (*P* < 0.0001). (B) Kaplan–Meier survival curves for patients stratified by sex (*P* < 0.0001).

The 5‐year OS rates according to patient age were 17.1% (<45 years), 17.9%, (45–64 years), and 14.1% (>=65 years). Patients over 65 years of age at the time of diagnosis (*n *=* *3576) had a significantly inferior 5‐year OS rate (Fig. 2A). Similar 5‐year survival and median survival rates were demonstrated for patients less than 45 years of age and patients 45 to 64 years of age.

**Figure 3:**
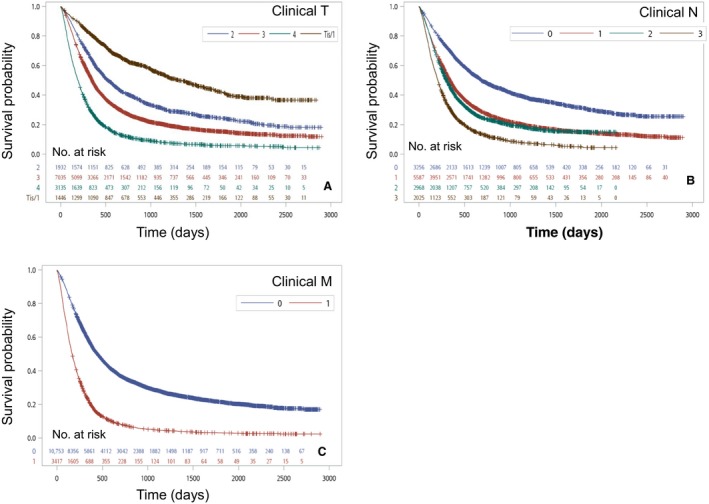
(A) Kaplan–Meier survival curves for patients stratified by clinical T stage (*P* < 0.0001). (B) Kaplan–Meier survival curves for patients stratified by clinical N stage (*P* < 0.0001). (C) Kaplan–Meier survival curves for patients stratified by clinical M stage (*P* < 0.0001).

Men diagnosed with esophageal cancer (*n *=* *13,558) had a worse prognosis than women (Fig. 2B). Women with esophageal cancer (*n *=* *836) had a longer median survival (391 days) and better 5‐year survival rate (23.11%) than men (median survival, 341 days; 5‐year survival rate, 16.4%; both, *P *<* *0.001). Figure 3A shows the distribution of patients by clinical “ T ”: Tis/T1 (*n *= 1446;10.0%), T2 (*n *= 1932;13.4%), T3 (*n *= 7035;48.9%), and T4 (*n *= 3135;21.8%). The 5‐year survival rates by clinical “ T ” were 41.0% for Tis/T1, 24.2% for T2, 15.2% for T3, and 5.7% for T4. The OS rates were significantly different (*P *<* *0.0001). A similar result for clinical “ N ” is shown in Figure 3B. The distribution of patients by clinical “ N ” is as follows: N0 (*n *= 3256; 22.6%), N1 (*n *= 5587; 38.8%), N2 (*n *= 2968; 20.6%), and N3 (*n *= 2025; 14.1%). The 5‐year survival rates by clinical “ N ” were 30.8% for N0, 14.9% for N1, 14.9% for N2, and 4.4% for N3. There was no significant difference of OS in N1 and N2, but differences were noted between N0, N1/N2, and N3 (*P *<* *0.0001). The survival curve was stratified by clinical “ M ” (Fig 3C). The 5‐year OS rate in M0 (21.3%) was better than that in M1 (3.0%). The survival time was longer in M0 (444 days) than in M1 (166 days) (*P *<* *0.0001).

**Figure 4:**
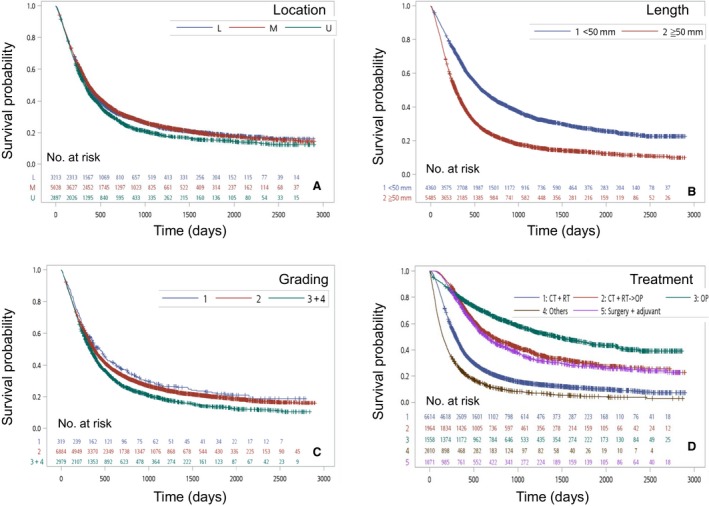
(A) Kaplan–Meier survival curves for patients stratified by tumor location (*P* < 0.0001). (B) Kaplan–Meier survival curves for patients stratified by tumor length (*P* < 0.0001). (C) Kaplan–Meier survival curves for patients stratified by tumor grading (*P* < 0.0001). (D) Kaplan–Meier survival curves for patients stratified by treatment method (*P* < 0.0001).

Turning to tumor location, patients with cancer in the upper third of the esophagus had a worse 5‐year OS rate (15.0%) than those with cancer in the middle third (18.4%) or lower third (19.1%) of the esophagus. A shorter survival time was also noted in the upper third (329 days) cases compared to the middle third (368 days) and lower third (364 days) cases (*P *<* *0.0001) (Figure 4A). As seen in Figure 4B, the 5‐year OS rate (27%) for patients with tumors less than 5 cm in length surpassed the 5‐year OS rate (13.2%) for patients with tumors at least 5 cm in length. The respective survival times are 588 days and 286 days; they are significantly different. Figure 4C shows the distribution of patients by histologic grade: G1 (*n *= 319; 2.2%), G2 (*n *= 6884; 47.8%), and G3 (*n *= 2979; 20.7%). The 5‐year survival rates by histologic grade were 21.8% for G1, 19.7% for G2, and 13.2% for G3; these rates were significantly different (*P *<* *0.0001).

The population database also contained the information about the initial treatment each patient received (generally within 3 months of diagnosis). However, there were no comprehensive data for all treatments recorded in the database. Concurrent chemoradiotherapy (CRT) was administrated to 6614 patients (45.9%), and CRT in combination with surgery was performed in 1964 patients (13.6%). According to the database analysis, 1558 patients (10.8%) received only surgical resection. Additionally, 1071 patients underwent surgical resection with adjuvant CRT (7.4%) and 2010 patient received other therapy methods (14.0%).

During the study period, patients who underwent surgical resection had the best 5‐year survival rate (44.8%). Patients who received CRT in combination with surgery and patients who underwent surgical resection with adjuvant CRT had 5‐year survival rates of 29.9% and 27.6%, respectively. Patients who underwent only CRT had a poorer 5‐year survival rate (10.6%). Patients who received other types of therapy had the worst 5‐year survival rate (4.6%) (Fig. 4D).

In univariate analysis, age, sex, tumor location, tumor length, histologic grade, clinical T, clinical N, clinical M, clinical stage, and treatment were found to be statistically associated with OS (Table 1). A multivariate Cox regression model was constructed incorporating patient age, sex, tumor location, tumor length, histologic grade, clinical T, clinical N, clinical M, and treatment methods. Age, sex, tumor location, tumor length, clinical T, clinical N, clinical M, and treatment remained independent prognostic factors (Table 2).

**Table 2:** Multivariate analysis of overall survival

Variables	HR	95% Confidence Interval	*P* value
Age			
<45 (reference)	1	‐	
45‐64	0.86	0.78‐0.96	0.0067
>=65	0.94	0.84‐1.06	0.3432
Sex			
Male (reference)	1	‐	
Female	0.83	0.71‐0.97	0.0191
Tumor location			
U/3 (reference)	1	‐	
M/3	0.94	0.87‐1.02	0.1116
L/3	0.87	0.80‐0.96	0.0048
Tumor length			
<5 cm	1	‐	
≧5 cm	1.25	1.16‐1.35	<0.0001
Grade			
G1	1	‐	
G2	0.86	0.72‐1.03	0.1103
G3/G4	0.93	0.77‐1.12	0.4376
Clinical T classification			
T1 (reference)	1	‐	
T2	1.45	1.24‐1.69	<0.0001
T3	1.91	1.64‐2.23	<0.0001
T4	2.79	2.36‐3.30	<0.0001
Clinical N classification			
N0 (reference)	1	‐	
N1	1.18	1.07‐1.30	0.0012
N2	1.27	1.14‐1.43	<0.0001
N3	1.41	1.24‐1.60	<0.0001
Clinical M classification			
M0 (reference)	1	‐	
M1	1.68	1.54‐1.83	<0.0001
Treatment			
CCRT	1	‐	
Only surgery	0.62	0.54‐0.70	<0.0001
CCRT + surgery	0.48	0.43‐0.53	<0.0001
Surgery + adjuvant	0.72	0.64‐0.81	<0.0001
Others	1.75	1.58‐1.94	<0.0001

HR, hazard ratio.

## Discussion

This study was a retrospective study investigating the clinic‐pathologic features of patients with esophageal cancer in Taiwan, based on the 7th AJCC staging system. Chen et al. reported that age, sex, and curative treatment were significant predictors of lifetime survival in patients with esophageal cancer 12. Our study indicated that not only the factors mentioned above but also tumor location, tumor length, clinical T, clinical N, and clinical M remained independent prognostic factors in multivariate analysis.

An increasing trend in the incidence of esophageal squamous cell carcinoma was noted 15. In Europe and America in 2006, the peak incidence by age (30%) of esophageal cancer patients was in those older than 75 years 16, 17. In Taiwan, most of the incidence of the esophageal cancer reported in 2013 was in patients 51–60 years of age 12. However, our study showed that most of the patients were diagnosed at ages between 45 and 64. The results revealed that the peak incidence by age decreased in recent years. Moreover, the 5‐year OS rate in the group 45–64 years old (17.9%) was significantly better than the 65 and over group (14.1%).

According to our results, the male/female esophageal cancer incidence ratio was 16.2. Moreover, males had significantly lower 5‐year survival rates and median survival time than females. Therefore, sex was a strong and independent prognostic factor. These findings were in accordance with past studies 12, 18. Our study showed that tumors located at the upper third of the esophagus predicted poorer outcomes than tumors located at the middle third and lower third. According to other studies 19, 20, this result might be due to the closeness of the upper third to the trachea and the high tendency to show proximal lymphatic spread bilaterally along the recurrent laryngeal nerve.

In the AJCC Cancer Staging Manual, 8th edition, the factor of tumor size is regarded as an independent predictor of prognosis 21. Our study revealed that tumors smaller than 5 cm were linked to significantly better survival outcomes than tumors larger than 5 cm.

Turning to the factor of clinical “N”, the accuracy of clinical N staging is questioned. The previous study showed that 40% of patients staged as clinical N0 went on to have pathologic N+ at surgery. Due to the inaccuracy of clinical N staging, the AJCC 6th edition only separated clinical N staging to N0 and N+ groups. Although the role of clinical N staging is not as important as pathologic N staging, Sheraz et al. 22 showed that the cN+pN0 patients have poor prognosis than cN0pN0 patients. The clinical N staging gets more important, and AJCC 7th edition separated clinical N staging to N0, N1, N2, and N3 groups. Our study found that there was no significant difference of OS in N1 and N2, but statistical differences were noted between the N0, N1/N2, and N3 groups.

Most of the patients in our study underwent only CRT (6614 patients, 45.9%), whereas only 4593 patients (32%) could receive surgical resection. The NCCN guidelines version 4.2017 recommended that patients with esophageal squamous cell carcinoma staging cT1b‐T4a / N0‐N+ or cT4b who medically unable to tolerate major surgery should receive definitive chemoradiation therapy (dCRT). Although several studies suggested that surgery after dCRT could improve the prognosis of these patients, there are two randomized clinical trials find that there is no significant benefit in adding surgery after chemoradiotherapy.

Bedenne et al. 23analyzed 259 patients with operable T3N0‐1 esophageal cancer. The two‐year survival rate was 40% in the dCRT group versus 34% in CRT‐S (*P* = 0.44).

Stahl et al. 24 studied 172 eligible randomized patients with locally advanced esophageal SCC (T3‐4N0‐1) who received either dCRT or CRT‐S. There was no significant difference of 2‐year and 3‐year overall survival rate between two groups.

Due to the controversial issue of these patients should receive further surgery or not, most of the patients only received dCRT at oncologist without further surgery intervention.

Our study showed that patients who underwent surgery alone had better outcomes than patients who underwent neoadjuvant CRT plus surgery or surgery plus adjuvant CRT. This result was due to patients who needed to undergo CRT having an advanced clinical stage at diagnosis. The guidelines of the National Comprehensive Cancer Network (NCCN) in 2015 recommended that surgery alone could be used at stage T1N0 and that neoadjuvant CRT should be used for patients in stages other than T1N0 25. Several studies reported that at the same diagnostic stage, the addition of CRT leads to a survival benefit compared to surgery alone. At clinical stage II/III of esophageal SCC, both neoadjuvant CRT and adjuvant CRT demonstrated a survival benefit compared with surgery alone 26. Rice et al. declared that the addition of postoperative adjuvant CRT after an esophagectomy could significantly improve survival time, time to recurrence, and recurrence‐free survival 27. In Taiwan, Hsu et al. 28 reported that for patients who had positive lymph nodes, the 3‐year OS rate was significantly higher in the surgery with adjuvant CRT group (45.8%) than in the surgery alone group (14.1%) in 2014.

Of the 14,394 patients diagnosed with esophageal cancer in our study, the 5‐year OS rate was 16.8%. In 2014, Anil et al. 28 reported that about 30% of esophageal cancers were found with regional metastasis and 40% with distant metastasis x. We found that there were only 25.4% of esophageal cancers diagnosed at stage IV.

There are some limitations of our study. First, the absence of some cancer‐specific data resulted in the inability to classify some patients; this could affect the data analysis. Second, due to data on cancer recurrence were not being available, only OS was used as a gauge. Third, our study was staged according to the 7th edition of the TNM staging system. The 8th edition provides a more detailed and more reasonable protocol for the staging of stage II and III esophageal cancer. Although there were several limitations, our findings still have important roles in the identification of prognostic factors and the prediction of survival outcomes.

In conclusion, the overall survival of esophageal cancer patients has been improving. Our data indicated that age, sex, tumor location, tumor length, clinical T, clinical N, clinical M, and treatment remained independent prognostic factors. Patients who could receive surgery had significantly better outcomes.

## Conflict of Interest

There is no conflict of interest
